# Transcriptomic and Metabolomic Investigation of Cell Wall Remodeling and Hormonal Signaling Dysfunction in Short Silique Mutant Rapeseed

**DOI:** 10.3390/ijms27188349

**Published:** 2026-09-19

**Authors:** Menglin Zhou, Xi Song, Bingbing Dai, Wei Zhou, Xiaofei Zan, Qijin Jing, Qingqing Yu, Wuming Deng

**Affiliations:** 1 Nanchong Academy of Agricultural Sciences, Nanchong 637000, China; 2 College of Agronomy and Biotechnology, Southwest University, Chongqing 400715, China

**Keywords:** *BnMYB46*, *B. napus*, multi-omics, phytohormones, short-silique mutant

## Abstract

Silique development is a critical factor influencing the yield of rapeseed (*B. napus*). This study employed the CRISPR/Cas9-mediated *BnMYB46* knockout mutant *K46* to investigate the molecular mechanisms underlying its short-silique phenotype via integrated cytological, transcriptomic, untargeted metabolomic, and targeted hormone metabolomic analyses. The *K46* mutant exhibited significantly reduced silique length, seed number per silique, and dry weight. Cytological observations revealed profound folding of epidermal cells and severe compression of parenchyma cells, consistent with impaired cell expansion. Multi-omics analysis identified plant hormone signal transduction as a core hub pathway disrupted at 20 DAF, and targeted metabolomics confirmed significant endogenous IAA and GA imbalances. qRT-PCR validation showed key hormone genes were significantly altered in *K46*, with decreases of 22.3-fold in *IAA2* and 18.8-fold in *GA3OX1* at 20 DAF, and a 165-fold increase in *ABF4*. These early hormonal disruptions drove downstream suppression of cell wall synthesis and carbohydrate metabolism pathways, leading to impaired cell expansion and compromised structural integrity. This study provides new insights into the hormonal regulatory network governing silique development and identifies potential targets for the molecular improvement of silique-related traits in rapeseed.

## 1. Introduction

Rapeseed (*B. napus* L.) is a vital oilseed crop in global agricultural production, with its seeds serving as a significant source of edible vegetable oil and high-quality plant protein. Additionally, rapeseed plays a crucial role in bioenergy and industrial feed sectors [1,2]. However, the current rapeseed industry in China is experiencing severe structural supply-demand imbalances, characterized by a substantial gap between domestic rapeseed production and the rapidly increasing processing demand. This discrepancy severely hampers the enhancement of vegetable oil self-sufficiency and the raw material security of downstream industries [3]. Consequently, analyzing the key factors that limit the yield potential of rapeseed and exploring its genetic regulatory mechanisms have become critical issues that urgently require breakthroughs in contemporary breeding science.

Silique length is a crucial agronomic trait that significantly influences the final seed yield of rapeseed, as it indirectly regulates yield levels by impacting both the number of seeds per silique and seed weight [4,5]. Being a complex quantitative trait governed by multiple genes, the population characteristics of silique length follow a normal distribution, which aids in the localization of quantitative trait loci (QTL) [6,7,8]. Current transcriptome analyses have demonstrated that a considerable number of genes associated with photosynthesis, hormone signal transduction, and secondary metabolite synthesis are highly expressed in the silique pericarp during critical developmental stages [9,10]. Currently, five functional genes that regulate silique development have been identified in rapeseed through cloning: *BnaA9.CYP78A9*, *BnaA09.ARF18*, *BnaC07.ROT3*, and *BnaC01.CCT8*, *BnaA05.DAD1*. These genes are recognized for their role in regulating silique length [11,12,13,14].

*MYB46*, a core transcription factor for secondary cell wall synthesis, exhibits a synergistic regulatory role in development and stress resistance across multiple species. In *Arabidopsis*, *MYB46* maintains cuticle integrity and mechanical strength by directly activating cuticle biosynthesis genes (such as *LACS*, *KCS*, and *CER*) and cell wall-associated genes. Recent research in *B. napus* has demonstrated that *BnaC9.MYB46* plays a pivotal role in drought tolerance by dually regulating cuticle and cell wall biosynthesis to maintain epidermal permeability [15]. In strawberries, CRISPR-Cas9-mediated knockout of *FvMYB46* results in a severe decline in pollen germination and fruit set rates, alongside a substantial reduction in lignin and flavonoid metabolites in flowers. Transcriptomic analysis reveals that *FvMYB46* positively regulates xylan biosynthesis, reactive oxygen species homeostasis, and the phenylpropanoid pathway, and is influenced by upstream NAC factors and the jasmonic acid (JA)-MYC2 module, thereby serving as a bridge between reproductive development and environmental response [16]. The loss of *MYB46* function not only leads to structural defects in the secondary wall but also triggers systemic disruptions in metabolic and hormonal networks. The physical collapse of the cell wall and impeded reproductive development resulting from *MYB46* mutation provide a crucial theoretical basis for understanding its role in the development of oilseed rape pods and the trade-off between yield and stress resistance.

Plant hormones, particularly indole-3-acetic acid (IAA), gibberellins (GA), and abscisic acid (ABA), play a crucial role in organogenesis, cell proliferation and expansion, as well as the maturation and senescence processes of fruits and siliques [9,17,18]. During the early stages of silique development, GA signaling promotes the degradation of DELLA repressor proteins, which alleviates their negative regulation on cell elongation and facilitates the longitudinal and transverse expansion of pod wall cells [19]. Simultaneously, IAA serves as an early initiator, establishing the groundwork for fruit set and cell division [20]. As siliques progress into the mid-development stage, the accumulation of ABA becomes increasingly dominant; it not only initiates dehydration and maturation programs but also halts further cell elongation through a strong antagonistic interaction with GA [21]. These three hormones do not function independently; instead, they are interconnected within a complex signaling network, creating a dynamic hormonal spatiotemporal balance that directly influences the final morphology of the silique.

Cell elongation fundamentally relies on the reversible loosening of the cell wall, a process mediated by various cell wall-modifying enzymes. Among these, the expansin family plays a crucial role in reducing the rigidity of the cell wall by disrupting the hydrogen bond network between cellulose microfibrils and hemicellulose. This disruption allows for the irreversible expansion of the cell, driven by turgor pressure [22]. However, the relaxation of the cell wall is not an isolated event; it is precisely regulated by upstream hormonal signals [23]. Crucially, while earlier studies have established a phenotypic association between hormones (IAA, GA, ABA) and cell elongation, the intricate mechanisms by which the loss of *BnMYB46* disrupts this hormonal dynamic balance—thereby impairing downstream cell wall remodeling and resulting in cell collapse—remain inadequately addressed through comprehensive cytological and multi-omics analyses.

To address this knowledge gap and systematically connect the molecular changes to the phenotypic defects, this study employed a multi-dimensional omics strategy specifically targeting the early developmental stage of the *K46* mutant. We applied transcriptomic sequencing (RNA-seq) to elucidate the gene expression signatures associated with hormonal signal transduction and cell wall restructuring, with the aim of uncovering the transcriptional cascades that drive the inhibition of cell elongation. Additionally, we utilized targeted hormonal metabolomics to quantitatively assess the levels of endogenous auxin, gibberellin, and abscisic acid, thereby providing corresponding chemical evidence for the observed transcriptomic discrepancies. Furthermore, we adopted untargeted metabolomics to globally profile metabolic shifts, particularly in carbohydrate and phenylpropanoid metabolism, which are anticipated to reflect the loss of turgor pressure and the depletion of structural precursors. By integrating these omics datasets with cytological observations and qPCR validation, we aimed to elucidate how the depletion of auxin/gibberellin signaling and the elevation of abscisic acid synergistically suppress cell wall loosening genes, ultimately leading to the inhibition of cell elongation and the physical collapse of silique cells. This research not only provides new insights into the molecular regulatory network governing silique length in rapeseed but also offers potential theoretical targets for enhancing high-yield traits in rapeseed through the manipulation of hormone signaling pathways.

## 2. Results

### 2.1. Identification and Cytological Observation of Short Silique Phenotype of K46 Mutant

**Figure 1:**
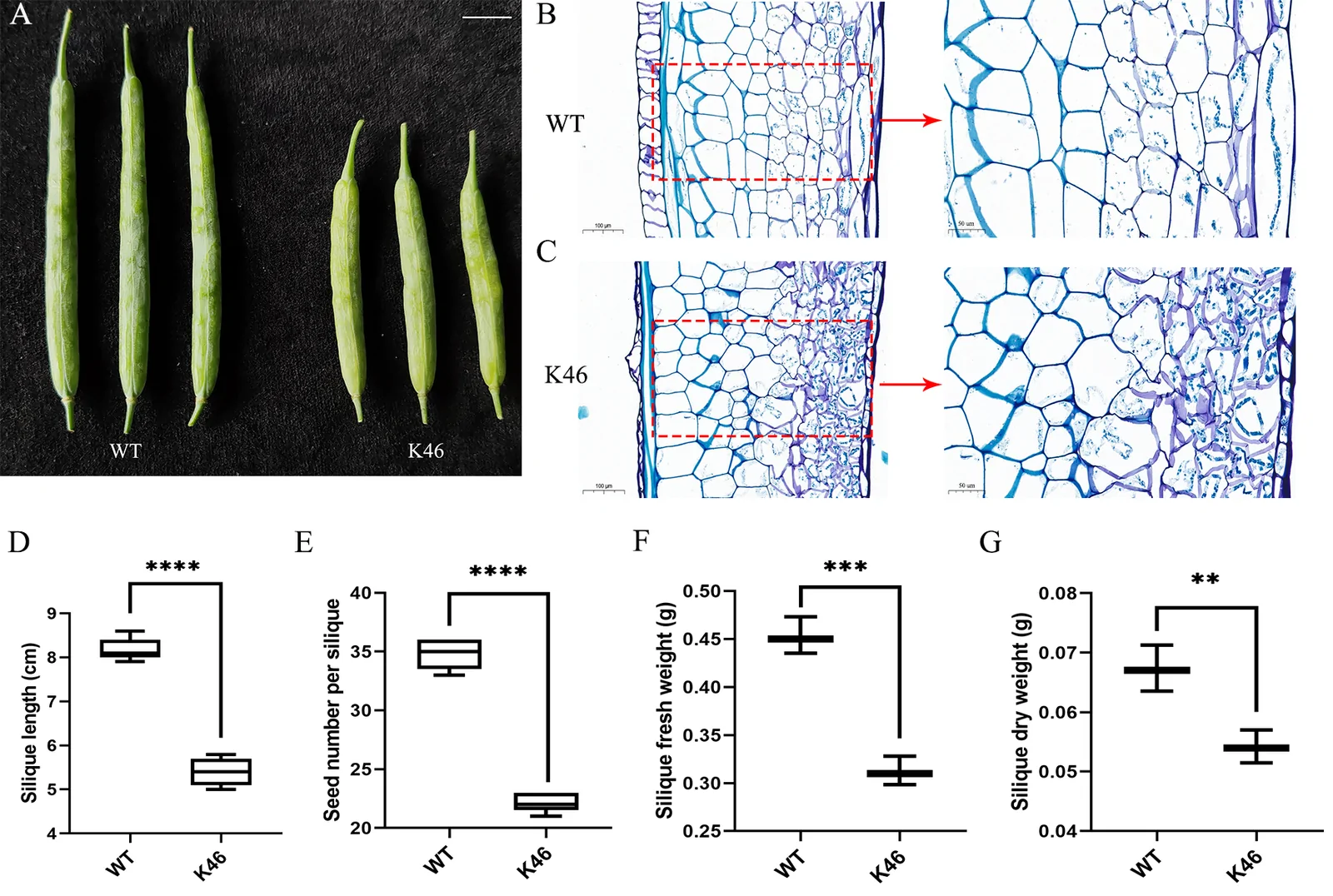
Phenotypic and cytological characteristics of WT and *K46* mutant siliques. (**A**) Morphological comparison of siliques between WT and *K46* mutant plants at the mature stage. (**B**,**C**) Toluidine blue-stained longitudinal sections of the silique wall. Red dashed boxes indicate the regions shown at higher magnification on the right, highlighting the epidermal and parenchymal cell morphology in WT (**B**) and *K46* (**C**) plants. (**D**) Silique length. (**E**) Seed number per silique. (**F**) Silique fresh weight. (**G**) Silique dry weight. Data are presented as mean ± SD (*n* = 5 biological replicates, with 10 siliques measured per replicate). Asterisks indicate significant differences between WT and K46 determined by unpaired Student’s *t*-test (**, ***, and **** indicate *p* < 0.01, *p* < 0.001, and *p* < 0.0001, respectively). Bar = 1 cm in panel (**A**); Bar = 50 µm in panels (**B**,**C**).

To elucidate the phenotypic differences between the WT and the mutant *K46*, we systematically compared key agronomic traits, including silique length, silique width, number of seeds per silique, fresh weight, and dry weight. Compared to the elongated siliques of WT, the *K46* mutant exhibited a distinctly shorter silique phenotype (Figure 1A). Quantitative analysis showed that the average silique length of WT was 8.16 ± 0.25 cm, whereas that of the *K46* mutant was significantly reduced to 5.39 ± 0.30 cm (Figure 1D). Statistical analysis revealed that the silique length, number of seeds per silique, fresh weight, and dry weight of *K46* were significantly lower than those of WT, while no significant difference was observed in silique width (Figure 1D–G and and Figure S1).

To further investigate the cellular basis of this shortened phenotype, cross-sections of the silique wall were prepared and stained with toluidine blue. Quantitative cytological analysis was conducted using SVS image processing software. The longitudinal lengths of 10 representative parenchyma cells were randomly measured for each genotype. Although no significant differences were observed in the number of cell layers and the total thickness of the silique wall between the WT and the *K46* mutant, our measurements indicated that the average longitudinal length of the 10 parenchyma cells in the *K46* mutant was significantly reduced to 354.26 ± 37.47 μm, compared to 479.11 ± 46.60 μm in the WT, representing a decrease of approximately 26.1%. This significant reduction in cell expansion, rather than a decrease in cell division, suggests that impaired cell elongation is a primary factor contributing to silique shortening. Microscopic examination indicated that in WT, the epidermal cells were arranged neatly, and the underlying parenchyma cells were fully expanded, exhibiting a typical honeycomb structure. In stark contrast, the *K46* mutant displayed severe morphological distortions characterized by deeply folded and wrinkled epidermal cells, as well as highly irregular parenchyma cells that were compressed inward and adhered closely, indicating significant structural disorganization and collapse of the parenchyma cells. These morphological features are consistent with impaired cell expansion (Figure 1B,C).

### 2.2. Transcriptome Analysis of Silique Development of K46 Mutant and Functional Enrichment of Differentially Expressed Genes

**Figure 2:**
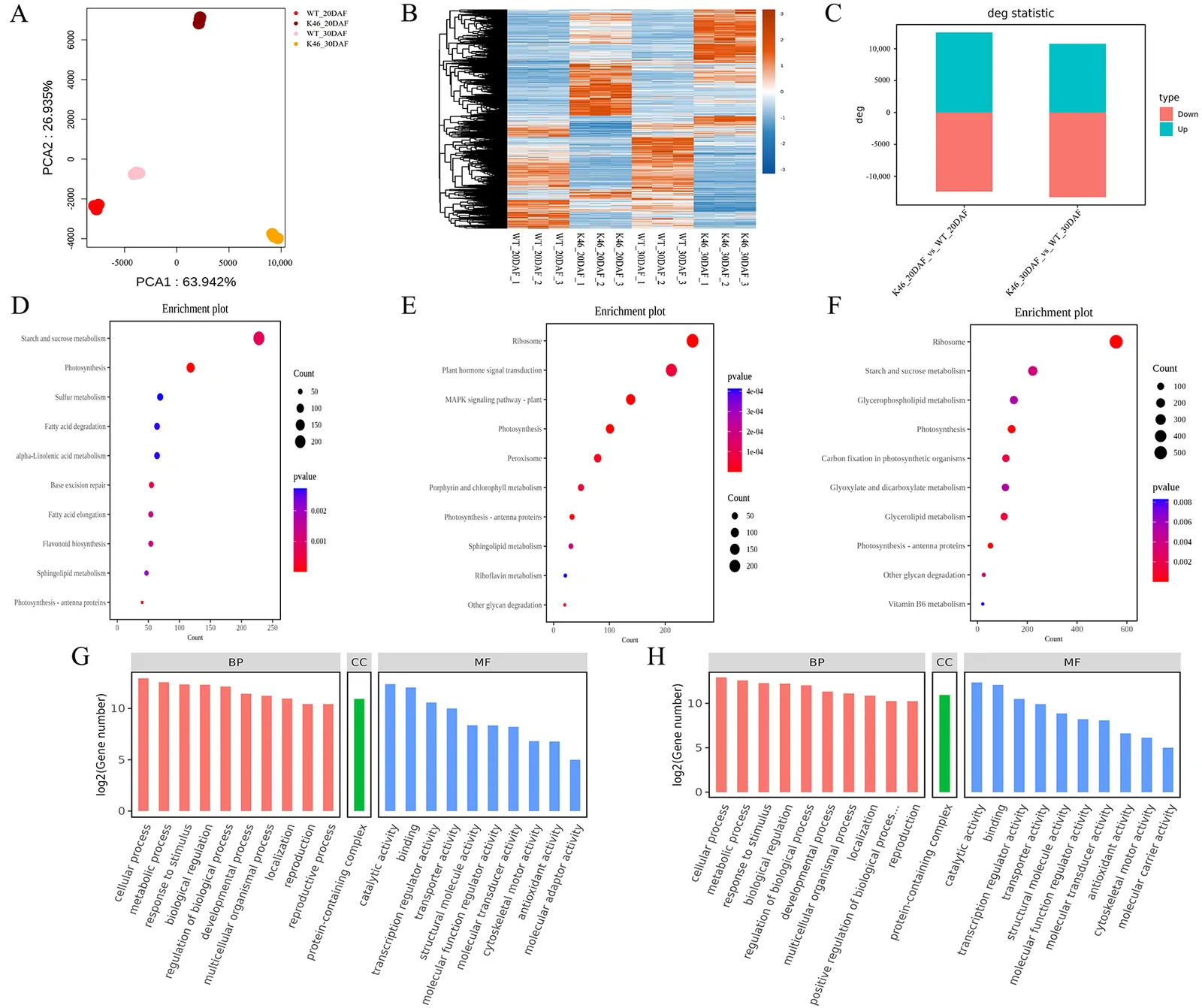
Transcriptomic profiling and functional enrichment analysis of WT and *K46* silique pericarps at 20 and 30 DAF. (**A**) PCA of transcriptome samples across the two developmental stages. (**B**) Heatmap illustrating the expression patterns of DEGs in WT and *K46* at 20 DAF and 30 DAF. (**C**) Statistics of up-regulated and down-regulated DEGs in the *K46*_20DAF_vs_WT_20DAF and *K46*_30DAF_vs_WT_30DAF comparison groups. (**D**–**F**) KEGG pathway enrichment analysis of DEGs. (**D**) Total DEGs at 20 DAF. (**E**) Down-regulated DEGs at 20 DAF. (**F**) Total DEGs at 30 DAF. Bubble size represents the number of enriched genes, and color scale indicates the *p*-value. (**G**,**H**) GO functional classification of DEGs across BP, CC, and MF categories at 20 DAF and 30 DAF.

To investigate the molecular basis of abnormal silique development in the mutant *K46*, we performed a transcriptomic analysis on the silique pericarp of both WT and *K46* at 20 and 30 DAF. Principal component analysis (PCA) revealed a significant influence of developmental timing on the transcriptome, with PC1 accounting for 63.94%. Hierarchical clustering heatmaps further confirmed substantial transcriptional reprogramming between WT and *K46* at these two critical developmental stages (Figure 2A,B). Differentially expressed gene (DEG) analysis indicated that in the comparison of WT_20DAF versus *K46*_20DAF, there were a total of 12,566 upregulated genes and 12,395 downregulated genes. In contrast, in the comparison of *K46*_30DAF versus WT_30DAF, there were 10,777 upregulated genes and 13,282 downregulated genes (Figure 2C).

To analyze the biological functions of DEGs, we conducted KEGG pathway enrichment analysis across various comparison groups. In the *K46*_20DAF_vs_WT_20DAF comparison group, the total DEGs were primarily enriched in pathways related to starch and sucrose metabolism, photosynthesis, fatty acid degradation, and linoleic acid metabolism (Figure 2D). Further enrichment analysis of downregulated DEGs during this period revealed a significant shift towards pathways involving ribosomes, plant hormone signal transduction, the MAPK signaling pathway, and photosynthesis (Figure 2E). Compared to the enrichment pattern of total DEGs, downregulated genes exhibited the highest gene abundance and significant enrichment in the ribosome and plant hormone signal transduction pathways. In the *K46*_30DAF_vs_WT_30DAF comparison group, DEGs were mainly enriched in pathways related to ribosomes, starch and sucrose metabolism, glycerophospholipid metabolism, photosynthesis, and carbon fixation (Figure 2F), with the ribosome pathway containing the highest number of differential genes, followed by the starch and sucrose metabolism and glycerophospholipid metabolism pathways.

Additionally, we conducted a functional classification of DEGs using Gene Ontology (GO) analysis. In the comparison groups *K46*_20DAF_vs_WT_20DAF (Figure 2G) and *K46*_30DAF_vs_WT_30DAF (Figure 2H), the patterns of GO enrichment distribution were notably consistent. At the Biological Process (BP) level, DEGs were predominantly enriched in cellular processes, metabolic processes, and responses to stimuli. In terms of Cellular Component (CC), genes associated with protein complexes exhibited significant enrichment. At the Molecular Function (MF) level, the primary categories included catalytic activity, binding, and transcription factor activity.

### 2.3. Metabolomics Analysis of Silique Development and Functional Enrichment of Differential Metabolites in K46 Mutant

**Figure 3:**
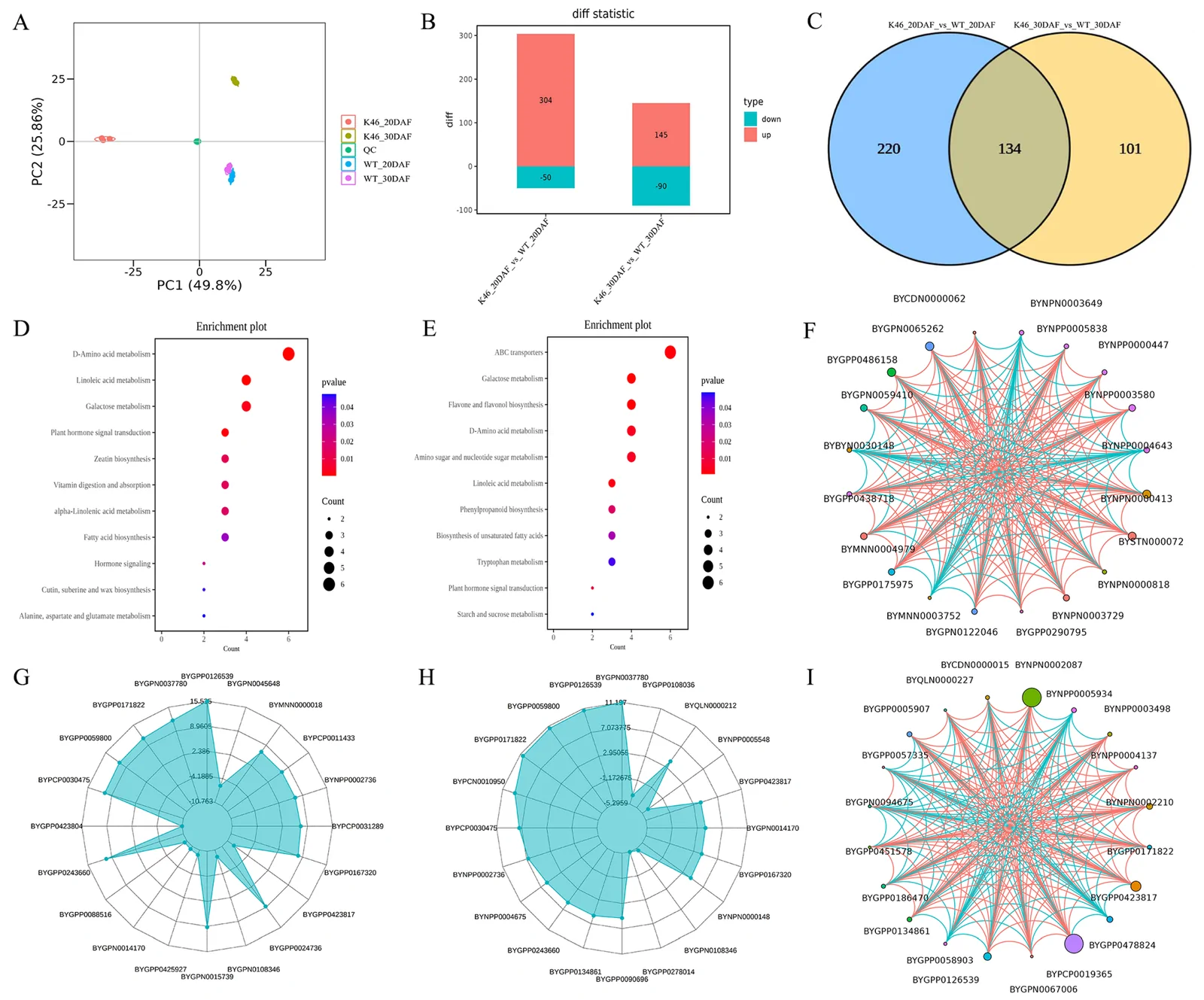
Metabolomic profiling and differential metabolite analysis of WT and *K46* siliques at 20 and 30 DAF. (**A**) PCA of metabolomic samples across the developmental stages. (**B**) Statistics of up-regulated and down-regulated differential metabolites in the *K46*_20DAF_vs_WT_20DAF and *K46*_30DAF_vs_WT_30DAF comparison groups. (**C**) Venn diagram showing the overlap and specific differential metabolites between the two comparison groups. (**D**,**E**) KEGG pathway enrichment analysis of differential metabolites at 20 DAF (**D**) and 30 DAF (**E**). Bubble size represents the number of enriched metabolites, and the color scale indicates the *p*-value. (**F**,**I**) Chord diagrams illustrating the correlation networks of key differential metabolites at 20 DAF (**F**) and 30 DAF (**I**), respectively. (**G**,**H**) Radar charts presenting the relative abundance distribution of significantly differential metabolites with high VIP scores at 20 DAF (**G**) and 30 DAF (**H**). The *K46*_20DAF, *K46*_30DAF, WT_20DAF, and WT_30DAF represent the pericarp samples from *K46* and WT siliques at 20 and 30 DAF, respectively; QC, quality control samples.

To analyze the metabolic differences between WT and *K46* at different developmental stages, a non-targeted metabolomics analysis was conducted on siliques sampled at 20DAF and 30 DAF. PCA revealed that the PC1 and the PC2 accounted for 49.8% and 25.86% of the total variance, respectively. The four sample groups (*K46*_20DAF, *K46*_30DAF, WT_20DAF, WT_30DAF) exhibited clear separation in the principal component space, with developmental stage (20 DAF_vs_30 DAF) serving as the primary source of variation along the PC1 axis, while genotype differences (WT_vs_*K46*) also effectively differentiated along the PC2 axis. Quality control samples (QC) clustered closely, indicating the stability of the analytical system (Figure 3A). Statistical analysis of differential metabolites identified a total of 354 differential metabolites in the *K46*_20DAF_vs_WT_20DAF comparison group, with 304 upregulated and 50 downregulated metabolites; in the *K46*_30DAF_vs_WT_30DAF comparison group, 235 differential metabolites were identified, with 145 upregulated and 90 downregulated (Figure 3B). Venn diagram analysis indicated that there were 134 overlapping differential metabolites between the two comparison groups, while the unique differential metabolites for the 20 DAF and 30 DAF periods were 220 and 101, respectively (Figure 3C).

To elucidate the functional distribution of differential metabolites, a KEGG pathway enrichment analysis was conducted on the aforementioned metabolites. In the *K46*_20DAF_vs_WT_20DAF comparison group, differential metabolites were significantly enriched in pathways such as D-amino acid metabolism, linoleic acid metabolism, galactose metabolism, plant hormone signal transduction, zeatin biosynthesis, vitamin digestion and absorption, α-linolenic acid metabolism, and fatty acid biosynthesis (Figure 3D). Among these, the pathways of D-amino acid metabolism, linoleic acid metabolism, galactose metabolism, and plant hormone signal transduction contained the highest number of differential metabolites and exhibited the greatest significance in enrichment. In the *K46*_30DAF_vs_WT_30DAF comparison group, differential metabolites were primarily enriched in pathways related to ABC transporters, galactose metabolism, flavonoid and flavonol biosynthesis, D-amino acid metabolism, amino sugar and nucleotide sugar metabolism, linoleic acid metabolism, phenylpropanoid biosynthesis, tryptophan metabolism, plant hormone signal transduction, and starch and sucrose metabolism (Figure 3E). Notably, the enrichment of ABC transporters, galactose metabolism, and flavonoid biosynthesis pathways was the most significant, with a high number of metabolites.

Further analysis through correlation network analysis reveals potential associations among core differential metabolites. Figure 3F,I present chord diagrams illustrating the significantly differential metabolites in the 20 DAF and 30 DAF comparison groups, respectively. These diagrams reflect the patterns of abundance variation and the interaction networks of differential metabolites across groups. Specifically, Figure 3F characterizes the strength of associations among core differential metabolites at the 20 DAF stage, while Figure 3I illustrates the correlation network among metabolites at the 30 DAF stage. Furthermore, a radar chart visualizes the relative distribution of key differential metabolites at different developmental stages. Figure 3G,H depict the relative abundance distribution characteristics and intergroup differences in significantly differential metabolites with high variable importance in projection scores (such as BYGPP0171822, BYCDN0000015, etc.) in WT and *K46* samples during the 20 DAF and 30 DAF stages, respectively.

### 2.4. Combined Transcriptome and Metabolomics Analysis

**Figure 4:**
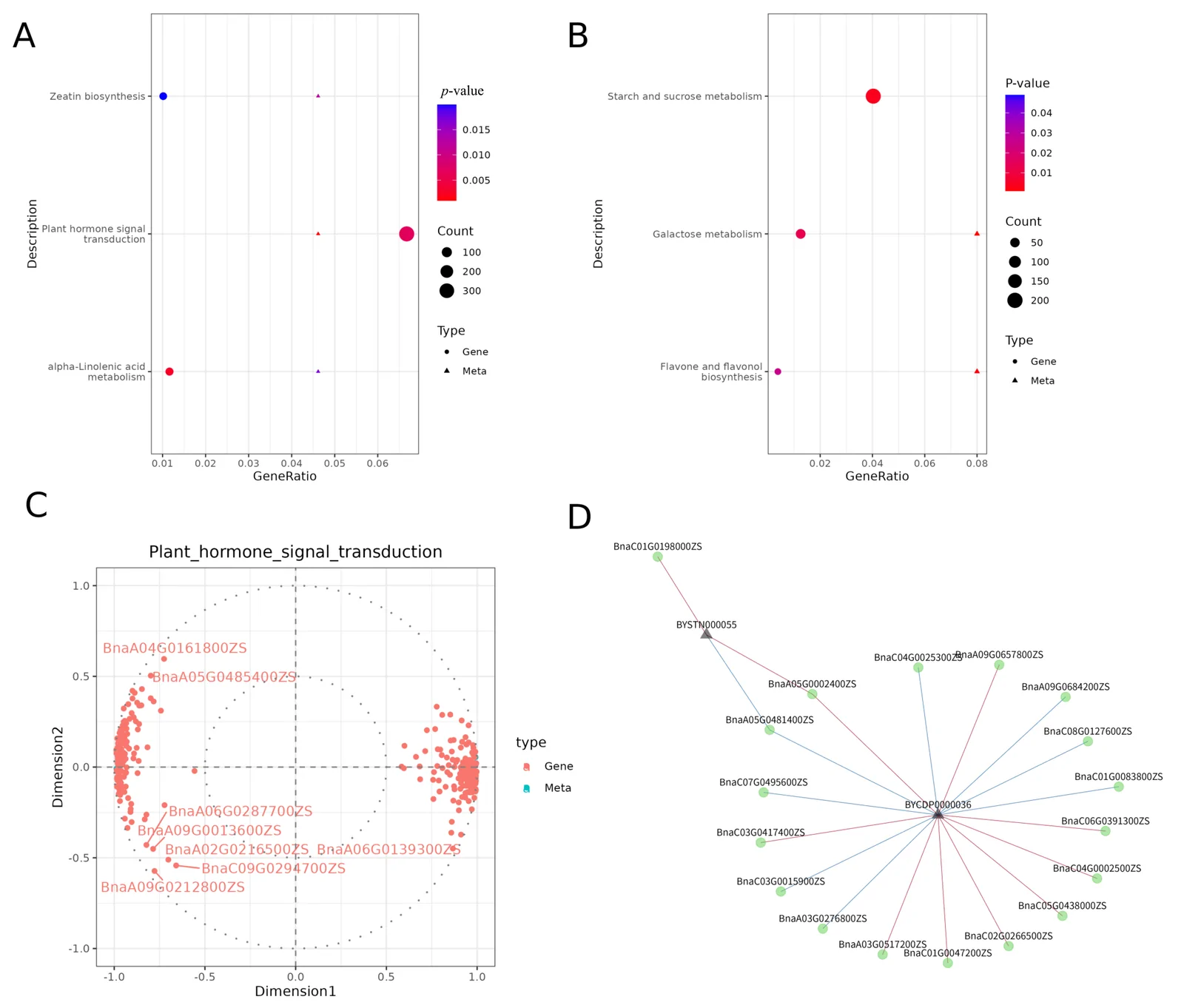
Integrated transcriptomic and metabolomic analysis of hormone-related regulatory networks in WT and *K46* mutant siliques. (**A**,**B**) KEGG pathway co-enrichment plots of DEGs and DEMs at 20 DAF and 30 DAF. Circle and triangle symbols represent genes and metabolites, respectively. (**C**) CCA of DEGs and DEMs involved in the plant hormone signal transduction pathway at 20 DAF. Red and blue dots indicate genes and metabolites, respectively. (**D**) Correlation network showing the interactions between key genes and metabolites within the plant hormone signal transduction pathway at 20 DAF. Circular and triangle nodes represent genes and metabolites, respectively; node size indicates the connectivity degree.

To further investigate the molecular regulatory mechanisms underlying the abnormal development of WT and *K46* siliques, this study conducted a comprehensive analysis utilizing transcriptomic and metabolomic data. By comparing the intersections of KEGG pathway enrichment results, the multi-omics analysis identified key metabolic pathways that were significantly co-enriched during two developmental stages. At 20 days after flowering (DAF), the pathways that were significantly co-enriched in both transcriptomic and metabolomic data included plant hormone signal transduction, zeatin biosynthesis, and alpha-linolenic acid metabolism. Notably, the plant hormone signal transduction pathway exhibited a pronounced co-enrichment at both the gene (Count > 200) and metabolite levels (Figure 4A). In contrast, at 30 DAF, the co-enriched pathways were primarily focused on starch and sucrose metabolism, galactose metabolism, and flavone and flavonol biosynthesis (Figure 4B).

Given the significant enrichment of plant hormone signaling pathways observed in the joint analysis at 20 DAF, a canonical correlation analysis (CCA) was performed on the differentially expressed genes and differentially abundant metabolites within this pathway. As illustrated in Figure 4C, during the 20 DAF period, several DEGs associated with plant hormone signaling (including *BnaA06G0161800ZS*, *BnaA05G0485400ZS*, and *BnaA09G0212800ZS*) demonstrated a notably dispersed distribution along the first dimension (Dimension 1) of the CCA ordination plot. This observation suggests that these hormone-related genes exhibit substantial transcriptional level variations between the wild type and the mutants, and the direction of these variations is well aligned with the trends of the corresponding metabolites.

The further construction of the correlation network between key genes and significantly different metabolites in this pathway is illustrated (Figure 4D). Network topology analysis indicates that during the 20 DAF period, metabolites related to plant hormones, such as BYCDN0000035 (Indole-3-Acetic Acid/IAA), occupy core hub nodes within the network. This metabolite demonstrates significant positive and negative associations with multiple genes involved in hormone signaling pathways (e.g., *BnaC01G019800ZS*, *BnaA09G0212800ZS*). Furthermore, the metabolites BYSTN0000055 (3-Indoleacetonitrile) and BYCDN0000100 (Jasmonic Acid) also exhibit direct interactions with the aforementioned gene nodes, indicating a synergistic expression network relationship. These results suggest that during the early developmental stage at 20 DAF, the regulatory network dominated by plant hormone-related metabolites plays a central role in the abnormal development process of the *K46* mutant silique.

### 2.5. Targeted Metabolic Profiling of Auxin, Gibberellin, and Abscisic Acid During Silique Development in the K46

**Figure 5:**
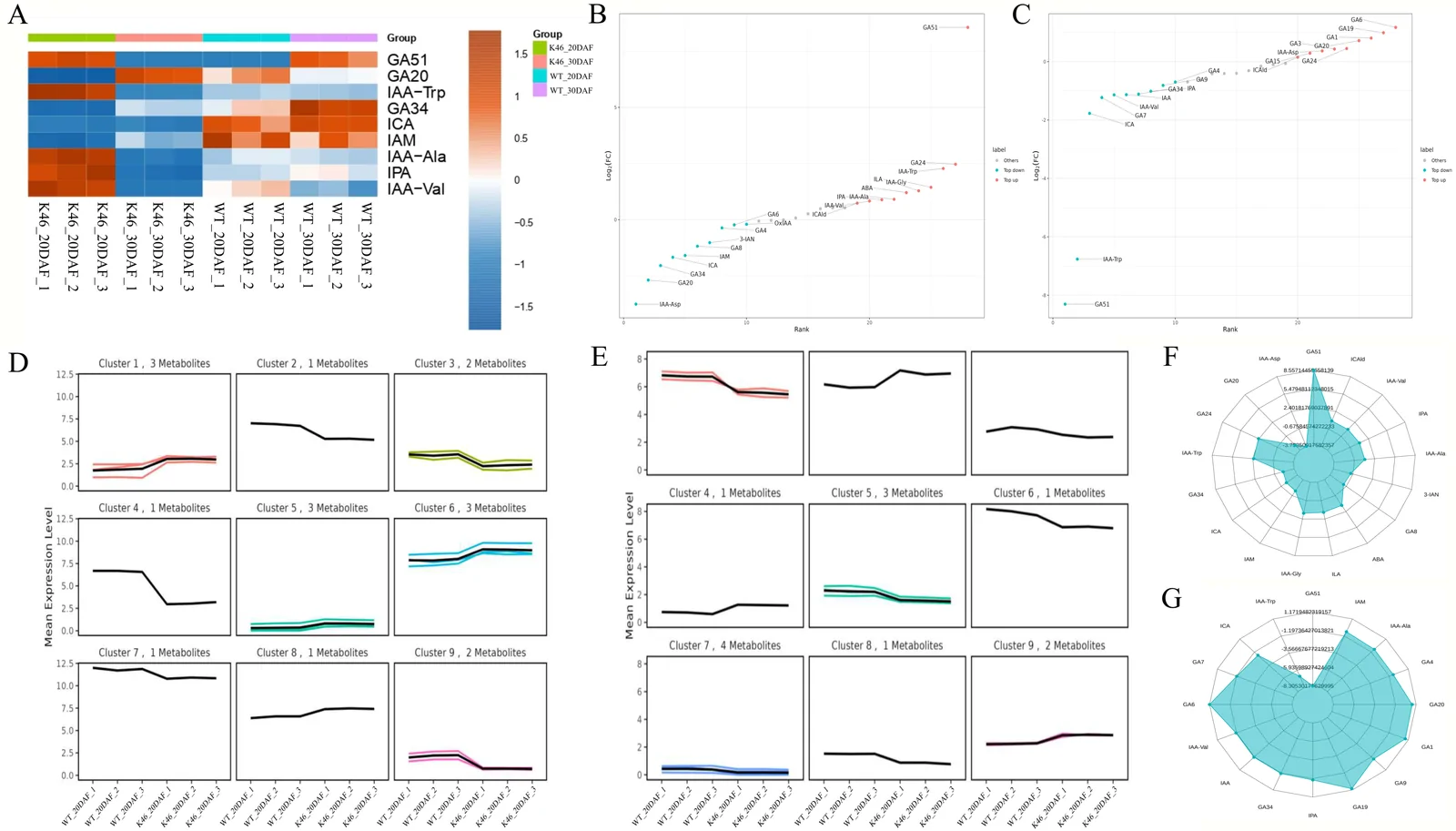
Targeted hormonal metabolomic profiling of auxin, gibberellin, and abscisic acid in WT and *K46* siliques at 20 and 30 DAF. (**A**) Heatmap showing the accumulation patterns of hormone-related metabolites across developmental stages. (**B**,**C**) Ranking distribution plots of differential metabolites with log_2_FC values at 20 DAF (**B**) and 30 DAF (**C**). Red and blue dots indicate up-regulated and down-regulated metabolites, respectively. (**D**,**E**) K-means clustering analysis of the accumulation patterns of differential hormone metabolites at 20 DAF (**D**) and 30 DAF (**E**). Different line colors indicate distinct clusters, with the name of metabolites in each cluster shown in Table S4. (**F**,**G**) Radar charts presenting the relative abundance of differential hormone metabolites at 20 DAF (**F**) and 30 DAF (**G**). The edge values indicate the mean accumulation levels.

To accurately analyze the endogenous hormonal regulation underlying the abnormal silique development of the mutant *K46*, we conducted targeted metabolomics detection of three classes of plant hormones: IAA, GA, and ABA, along with their related metabolites. The hierarchical clustering heatmap visually illustrates the changes in abundance of IAA, GA, and ABA-related metabolites at different developmental stages in both the WT and the *K46*. The heatmap reveals significant differences in the hormone metabolic profiles of the two materials at the same developmental stage, with certain gibberellins (e.g., GA51) and auxin precursors (e.g., IAA-Trp) showing high abundance accumulation in *K46* at 20 DAF, while the accumulation patterns of several metabolites reversed at 30 DAF (Figure 5A and Figure S2). The dynamic changes were further elucidated by the differential metabolite distribution plots at 20 DAF and 30 DAF. At 20 DAF, GA24, ILA, IPA, and IAA-Val were significantly upregulated, whereas GA51, IAA-Asp, and GA20 were significantly downregulated (Figure 5B). After entering 30 DAF, a notable shift in differential distribution occurred, with metabolites such as IAA-Trp, GA51, IAA-Val, and GA9 exhibiting a strong downregulation trend, while gibberellin precursors like GA19, GA1, and GA20 showed a relatively upregulated trend (Figure 5C).

The K-Means clustering algorithm was employed to categorize metabolites into nine distinct clusters based on the expression patterns of WT and *K46* at 20 DAF and 30 DAF. At 20 DAF, Cluster 1 encompasses GA24 and ILA, with accumulation levels in *K46* consistently higher than those in WT; Cluster 5 consists of IAA-Trp and GA51, which exhibit markedly elevated levels in *K46*; Cluster 9 includes GA20 and GA34, demonstrating significantly lower levels in *K46* compared to WT; Cluster 6 comprises IAA-Gly, ABA, and ICAld, which are slightly elevated in *K46* relative to WT; Clusters 4, 7, and 8 include 3-IAN, IAA-Ala, and IAA-Asp, each displaying unique accumulation patterns (Figure 5D and Table S6). At 30 DAF, the clustering pattern undergoes substantial alterations. Cluster 1 contains ICA and IAA-Ala, with accumulation levels in *K46* being lower than in WT; in Cluster 7, GA51, IAA-Trp, IAA-Val, and GA9 experience a sharp decline to very low levels in *K46*; Cluster 9 includes GA1 and GA20, which are significantly elevated in *K46* compared to WT; Cluster 5 comprises GA34, IPA, and GA4, showing reduced levels in *K46* compared to WT; and in Cluster 6, IAA is slightly lower in *K46* than in WT (Figure 5E and Table S6).

The relative content of various hormone-related metabolites in *K46* and WT at 20 DAF and 30 DAF was visually compared using radar charts. At 20 DAF, metabolites such as IAA-Val, GA24, ILA, and IPA were significantly more accumulated in *K46*, exhibiting a pronounced outward peak (Figure 5F). In contrast, at 30 DAF, the polygonal shape of the radar chart underwent significant changes, with the differential distribution characteristics of metabolites such as IAA, GA8, and GA19 between the two groups revealing distinctions across different dimensions. This indicates that with the development of the silique, the hormone metabolic profile in *K46* experienced significant temporal reprogramming (Figure 5G).

### 2.6. Integrative Correlation Analysis of Transcriptome and Targeted Hormonal Metabolome

**Figure 6:**
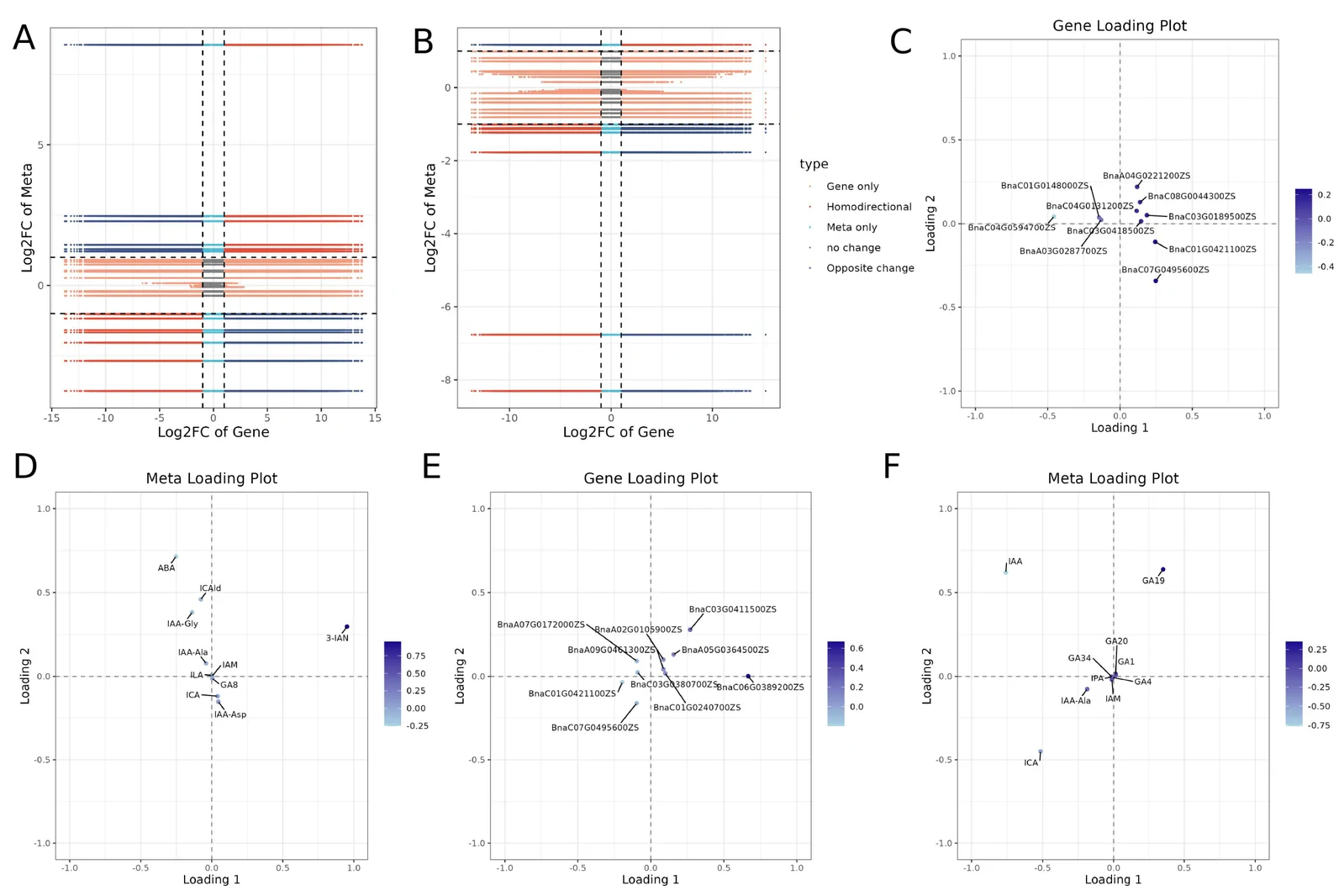
Joint analysis of transcriptome and targeted hormonal metabolome in WT and *K46* siliques at 20 and 30 DAF. (**A**,**B**) Nine-quadrant plots showing the Pearson correlation between gene expression levels and hormone metabolite abundance at 20 DAF (**A**) and 30 DAF (**B**). The dashed lines indicate thresholds of |*r*| = 0.8. (**C**–**F**) Principal component loadings plots of genes (**C**,**E**) and hormone-related metabolites (**D**,**F**) at 20 DAF (**C**,**D**) and 30 DAF (**E**,**F**). The *x*- and *y*-axes indicate the loadings on PC1 and PC2, respectively.

To further elucidate the hormonal regulatory mechanisms underlying the abnormal fruit development of the mutant *K46*, we conducted a comprehensive correlation analysis of transcriptomic data alongside targeted hormonal metabolomic data, specifically focusing on auxins, gibberellins, and abscisic acid. Utilizing the Pearson correlation coefficient, we constructed nine quadrant plots for 20 days after flowering (DAF) (Figure 6A) and 30 DAF (Figure 6B) to visually illustrate the interplay between transcript levels and the accumulation of endogenous hormonal metabolites. At 20 DAF, a significant number of genes demonstrated a strong positive correlation (positioned in the upper right or lower right quadrants) or a strong negative correlation (positioned in the upper left or lower left quadrants) with hormonal metabolites. Notably, the scatter points densely populated in quadrants 1, 3, 5, and 7 indicate that changes in gene expression levels are significantly coordinated or antagonistic to the abundance of hormonal metabolites (Figure 6A). At 30 DAF, while a considerable number of gene-metabolite pairs remained correlated, the distribution pattern of scatter points differed from that observed at 20 DAF, suggesting a dynamic evolution of the hormonal regulatory network across the two developmental stages (Figure 6B).

To identify the dominant variables driving the differences among groups, a principal component loading analysis was performed on transcriptomic data from two developmental stages, complemented by targeted hormone metabolomic data. In the gene loading plot for the 20 DAF stage, multiple genes involved in hormone signal transduction and cell wall synthesis exhibited high loading weights on PC1 and PC2. This observation indicates that these genes are the primary contributors to the transcriptional differences between WT and *K46* at 20 DAF (Figure 6C). Correspondingly, the metabolite loading plot for the 20 DAF stage revealed that several targeted hormone metabolites, including auxin precursors such as IAA-Trp, IAA-Asp, and gibberellins GA20 and GA34, also exhibited high absolute loading values. This suggests that these metabolites play a significant role in the intergroup differences at 20 DAF (Figure 6D). In the gene loading plot (Figure 6E) and metabolite loading plot (Figure 6F) for the 30 DAF stage, the dominant variables driving the differences changed, with a notable shift in the loading center at both the gene and hormone metabolite levels. For instance, the metabolite loading plot indicated that the loading weights of gibberellin synthesis precursors GA19, GA20, and ABA, which contributed less in earlier stages, increased relatively, suggesting a temporal transition in the types of metabolites that dominate hormonal regulation as silique development progresses into later stages.

### 2.7. qRT-PCR Validation of Key Differentially Expressed Genes in Hormone Signaling Pathways

**Figure 7:**
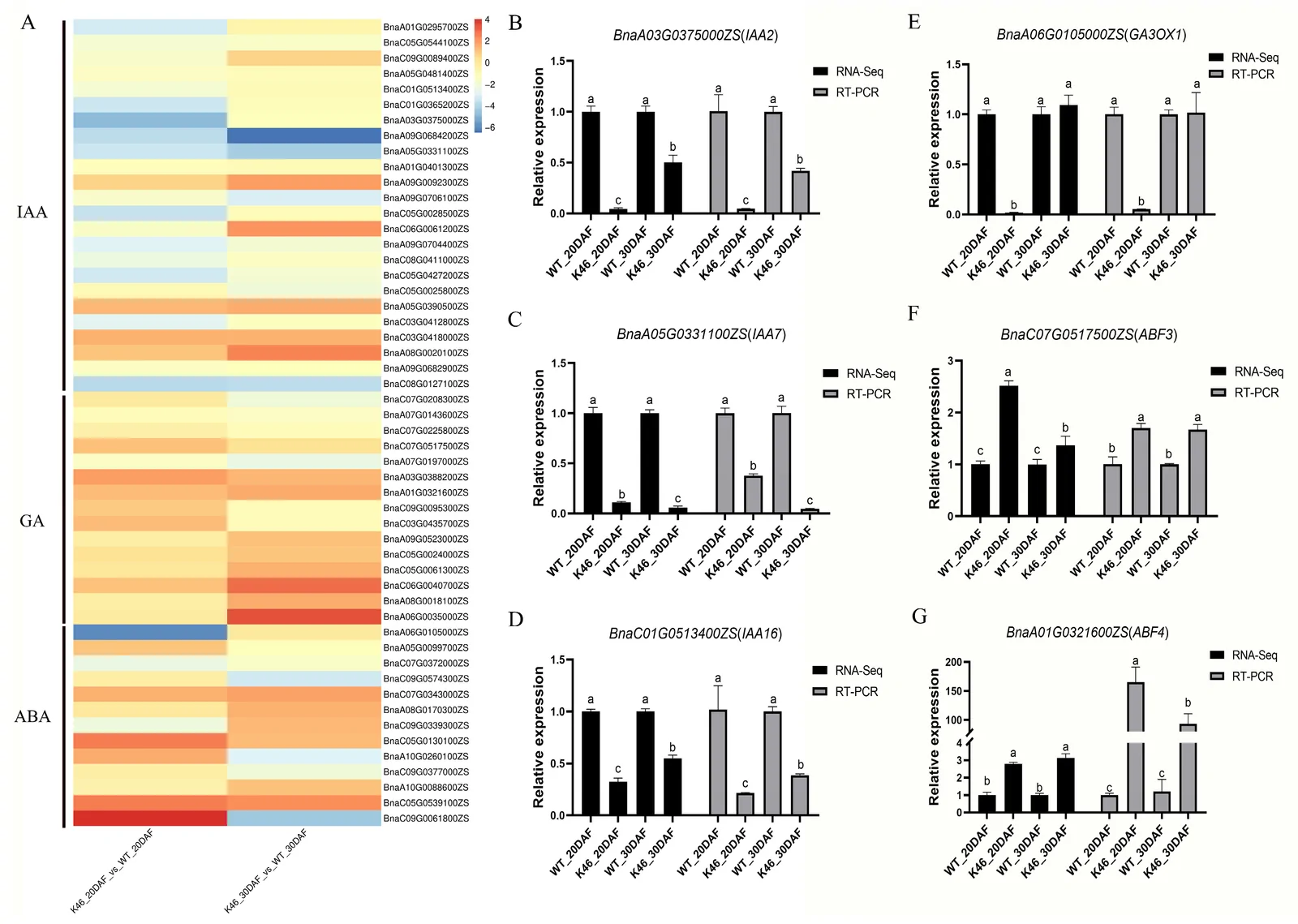
Validation of transcriptomic data by qRT-PCR for hormone-related genes. (**A**) Heatmap showing the expression changes (log_2_FC) of selected IAA, GA, and ABA related DEGs in K46_20DAF_vs_WT_20DAF and K46_30DAF_vs_WT_30DAF comparison groups. (**B**–**G**) Comparison of relative expression levels between RNA-seq and qRT-PCR for six selected genes: *IAA2* (**B**), *IAA7* (**C**), *IAA16* (**D**), *GA3OX1* (**E**), *ABF3* (**F**), and *ABF4* (**G**). Data are presented as mean ± SD, with three biological replicates. Different lowercase letters indicate significant differences among samples at *p* < 0.05.

To validate the reliability of the transcriptome sequencing data and further confirm the expression changes in hormone signal-related genes in the mutants, six key DEGs from the IAA, GA, and ABA signaling pathways were selected for qRT-PCR verification. Figure 7A visually presents the expression differences (log_2_FC) of the selected IAA, GA, and ABA-related differential genes between the two comparison groups: *K46*_20DAF_vs_WT_20DAF and *K46*_30DAF_vs_WT_30DAF. Figure 7B–G provides a detailed comparison of the relative expression results from RNA-seq and qRT-PCR.

Among the auxin signaling pathway-related genes, *BnaA03G0375000ZS* (*IAA2*) was significantly downregulated in *K46* at both stages, showing a 22.3-fold decrease in RT-PCR at 20 DAF and a 2.4-fold decrease at 30 DAF (Figure 7B). *BnaA05G0331100ZS* (*IAA7*) exhibited a 2.7-fold decrease at 20 DAF and a 21.5-fold decrease at 30 DAF (Figure 7C). *BnaC01G0513400ZS* (*IAA16*) showed a 4.7-fold decrease at 20 DAF and a 2.6-fold decrease at 30 DAF (Figure 7D). For the gibberellin biosynthesis gene, *BnaA06G0105000ZS* (*GA3OX1*) exhibited dramatic expression suppression in *K46* at 20 DAF, with an 18.8-fold decrease in RT-PCR. Notably, its expression recovered to levels comparable to WT at 30 DAF (Figure 7E). In the abscisic acid signaling pathway, *BnaC07G0517500ZS* (*ABF3*) was significantly upregulated in *K46*, showing a consistent 1.7-fold increase in RT-PCR at both 20 DAF and 30 DAF (Figure 7F). Conversely, *BnaA01G0321600ZS* (*ABF4*) exhibited a robust upregulation in *K46*, with an extraordinary 165-fold increase at 20 DAF and a 78-fold increase at 30 DAF in RT-PCR (Figure 7G).

In summary, the results of qRT-PCR validation are highly consistent with those obtained from transcriptome sequencing, thereby confirming the reliability of the transcriptome data presented in this study. Furthermore, these findings substantiate that the *K46* mutant exhibits significant transcriptional disturbances in the auxin, gibberellin, and abscisic acid signaling pathways during the early stages of silique development.

## 3. Discussion

As a core transcriptional regulatory factor in the synthesis of plant secondary cell walls, *MYB46/MYB83* has been confirmed to regulate the deposition of lignin and cellulose in *Arabidopsis* [24,25,26]. Recent studies in *B. napus* have revealed that *BnaC9.MYB46* is not only involved in cell wall synthesis but also plays a crucial role in maintaining epidermal permeability by precisely regulating cuticle-related genes, thus conferring drought resistance to plants [15]. However, direct evidence regarding the biological function of this gene in the morphological development of reproductive organs (siliques) in *B. napus* remains scarce, particularly concerning how its mutation results in macroscopic growth defects.

In this study, the CRISPR/Cas9-mediated *BnMYB46* gene-edited mutant *K46* exhibits a severe short silique phenotype, characterized by a significant reduction in seed number per silique and dry weight. Cytological microscopic observations reveal a clear pathological basis: the epidermal cells of the *K46* silique skin show deep folding, while the underlying parenchyma cells are severely collapsed and have lost turgor pressure. While this collapsed phenotype bears a strong resemblance to the vascular tissue development defects and cell wall collapse observed in the *Arabidopsis myb46* mutant, it is crucial to emphasize that this similarity is primarily phenotypic. The direct evidence from the present study confirms the loss of structural integrity and turgor in *B. napus* siliques. However, whether the underlying molecular mechanisms driving this collapse are identical across species is inferred from previous studies in *Arabidopsis* [26,27]. This hypothesis of evolutionary conservation is further supported by the contrasting phenotype observed in *BnaC9.MYB46*-overexpressing plants, which exhibited improved water retention attributed to increased cuticle coverage [15]. This “positive and negative verification” strongly suggests that *BnMYB46* not only regulates drought resistance in rapeseed but also plays a critical role in maintaining cell wall and cuticle integrity in reproductive organs. It should be noted that this study utilized a single homozygous *K46* line, whose homozygous status and stable phenotype were confirmed by Sanger sequencing and across generations. Although a single line represents a limitation, the consistent phenotype across independent biological replicates supports the role of *BnMYB46*. Future studies using additional independent lines will further validate these findings.

Multi-omics joint analysis reveals upstream triggering mechanisms. During the critical period of silique elongation at 20 DAF, the plant hormone signaling pathway was identified as a core hub co-enriched in transcriptomics and metabolomics [18,28,29]. Targeted hormone metabolomics and CCA further confirmed that the *K46* mutant exhibited a significant imbalance in endogenous IAA, GA, and ABA homeostasis at an early stage. Notably, auxin and gibberellin are classic signaling molecules that regulate cell expansion and cell wall loosening [30,31]. The absence of *BnMYB46* resulted in a marked downregulation of hormone-responsive and biosynthetic genes such as *IAA2*, *IAA7*, and *GA3OX1*, aligning with reports in plants regarding the regulation of *MYB46* by plant hormone response factors [32,33]. However, this specific crosstalk mechanism between *MYB46* and hormone signaling pathways is inferred from prior reports, while this study extends this regulatory axis into the spatiotemporal dimension of rapeseed silique development for the first time.

By integrating transcriptomic and metabolomic enrichment analyses, we further elucidated the downstream signaling pathways associated with hormonal dysregulation. In *K46*, pathways related to the cell wall, including ‘starch and sucrose metabolism’ and ‘phenylpropanoid biosynthesis’, exhibited highly significant enrichment, which directly reflects the transcriptomic and metabolomic changes observed in the present study. The significant downregulation of CAD, XTH, and PAL genes at 20 DAF provides direct molecular evidence for the severe collapse of parenchyma cells and deep folding of epidermal cells observed in the K46 mutant. These findings indicate that the loss of *BnMYB46* not only disrupts hormonal signals but also directly suppresses the cell wall loosening machinery, thereby impairing cell expansion and contributing to the compromised structural integrity observed in the mutant (Figure S3). The role of *MYB46* in regulating cellulose and lignin synthesis has been previously established in *Arabidopsis* [25,34,35]; therefore, inferring that this transcriptional regulation directly drives the observed changes in *K46* is based on previous studies. More importantly, the deeply folded epidermal cells in *K46* suggest potential defects in cuticle synthesis. Coupled with the observation that overexpression of *BnaC9.MYB46* enhances cuticle coverage, we speculate that the loss of the *BnMYB46* gene may result in decreased mechanical strength of the cell wall (collapse of parenchyma cells) and compromised structure of the epidermal cuticle (wrinkling of epidermal cells) [15]. However, direct biochemical measurements of cell wall components and cuticle content in the *K46* mutant are needed to comprehensively confirm this dual-synergistic defect. The maintenance of turgor pressure depends on a robust cell wall and an effective epidermal barrier that isolates the external environment [36,37]. The disruption of carbohydrate metabolism in *K46* may also lead to a deficiency of osmotic regulatory substances, further limiting the capacity to maintain turgor pressure. The current study directly provides the evidence for metabolic deficiencies, while the precise causal link to physical collapse is inferred from physiological principles observed in model plants [12,18].

In summary, this study outlines the functional panorama of *BnMYB46* in rapeseed from the perspective of reverse (*K46* silique phenotype). This transcription factor serves not only as the “master switch” for the dual pathways of cuticle and cell wall synthesis under drought stress but also as a core safeguard for maintaining the normal morphology of rapeseed siliques. This finding provides important theoretical guidance for the genetic improvement of silique-related traits in rapeseed. When utilizing the superior alleles of *BnMYB46* for drought-resistant variety selection, careful assessment of their potential impacts on silique development and yield traits is essential. In the future, screening for precise weak allelic mutations or employing promoter-specific expression regulation at specific developmental stages (such as 20 DAF) to balance the conflict between “stress resistance” and “yield (silique length)” will be a key breakthrough for the practical application of the *MYB46* gene in molecular breeding of rapeseed [38,39].

## 4. Materials and Methods

### 4.1. Plant Materials and Growth Conditions

This study employs the cabbage-type rapeseed variety Westar as the WT and the short silique mutant *K46* as the experimental material. The *K46* mutant was generated through targeted editing of the *BnMYB46* gene using CRISPR/Cas9-mediated gene editing technology and was confirmed as a stable homozygous mutant strain through Sanger sequencing within the genetic background of Westar. In this study, the CRISPR/Cas9-mediated mutant *K46* was generated by targeting multiple *MYB46* homologous genes in *B. napus*, and we collectively refer to the edited gene as *BnMYB46* here (Table S8). All plants were cultivated in pots (volume, 30 L; diameter, 32 cm) filled with a growth substrate composed of a 1:1 (*v*/*v*) mixture of commercial nutrient soil and field soil. Plants were grown in a naturally lit greenhouse under a natural photoperiod (spring and autumn), with a day/night temperature regime of 22–28 °C/15–18 °C and a relative humidity of 60–70%. Standard field management practices, including irrigation and pest control, were applied uniformly to all plants. For the transcriptomic and metabolomic analyses, three independent biological replicates were collected, and for each biological replicate, siliques from three individual plants were pooled to minimize individual variation. For silique collection, flowers on the main inflorescence were tagged on the day of full anthesis (when the flowers were completely open and pollen was shed), which was defined as 0 DAF. Siliques were subsequently harvested at 20 and 30 DAF from the tagged main inflorescences, immediately frozen in liquid nitrogen, and stored at −80 °C for subsequent analyses.

### 4.2. Phenotypic and Cytological Observations

In the phenotypic analysis, mature siliques were randomly collected from 5 individual plants (biological replicates) for each genotype, and 10 siliques were sampled from the main inflorescence of each plant. Measurements were taken for silique length (cm), silique width (cm), number of seeds per silique, fresh weight (g), and dry weight (g). For cytological observation, longitudinal sections of the silique walls were prepared from siliques collected 30 DAF. Following staining with 0.1% (*w*/*v*) toluidine blue, the microstructure of the epidermis and parenchyma cells was observed and photographed using an optical microscope (Olympus BX53, Evident Corporation, Tokyo, Japan) [40,41].

### 4.3. Transcriptomic Analysis

Total RNA was extracted from the pericarp of wild-type and mutant *K46* siliques at 20 and 30 DAF using the Plant RNA Extraction Kit, with three biological replicates for each sample. cDNA libraries were constructed and sequenced on the Illumina NovaSeq 6000 platform. Raw reads were processed with fastp to eliminate adapter sequences, low-quality bases, and reads shorter than 16 bp, resulting in clean reads. Clean reads were aligned to the ZS11.v0 reference genome of *B. napus* using HISAT2 with default parameters, retaining only uniquely mapped reads for subsequent quantification. Sequencing depths for all samples ranged from 5.43 to 6.67 Gb of clean bases, with Q20 scores above 99.60%, Q30 scores ranging from 97.68% to 97.95%, and GC content ranging from 44.49% to 46.74%. To evaluate biological reproducibility, Pearson correlation coefficients were calculated among the three biological replicates within each group, all of which exceeded 0.95 (typically >0.99). Gene expression levels were calculated using featureCounts; the resulting raw count matrix was used as the direct input for downstream differential expression analysis, while FPKM values were exclusively used for data visualization. DEGs between the WT and *K46* groups were identified using the DESeq2 R package based on the raw count matrix, with thresholds set at an absolute fold change ≥2 (|log_2_(Fold Change)| ≥ 1) and a false discovery rate (FDR) < 0.05 (calculated using the Benjamini–Hochberg method). GO and KEGG enrichment analyses of DEGs were conducted using Fisher’s Exact Test, with the significance threshold set at FDR < 0.05 [42,43,44].

### 4.4. Untargeted Metabolomic Analysis

Non-targeted metabolic profiling analysis was conducted on the pericarp samples of both wild type and mutant *K46* at 20 and 30 DAF, utilizing three biological replicates for each sample. A 70% methanol solution containing 0.5 µg/mL of 2-chloro-L-phenylalanine served as the internal standard for metabolite extraction from 100 mg of ground tissue. Following vortexing, ultrasonication, and centrifugation, the supernatant was filtered through a 0.22 µm membrane to prepare for LC-MS analysis. Quality control (QC) samples were created by mixing equal portions from each sample to ensure system stability during the analysis [45].

LC-MS analysis was conducted utilizing an ultra-high-performance liquid chromatography (UHPLC) system coupled with the Orbitrap Exploris 120 mass spectrometer (Thermo Fisher Scientific, Waltham, MA, USA). Separation was achieved using a Thermo Hypersil Gold C18 column (2.1 × 100 mm, 1.9 µm) maintained at a temperature of 40 °C with a flow rate of 0.3 mL/min. The mobile phase comprised a 5 mM ammonium acetate aqueous solution (Phase A) and acetonitrile (Phase B). The mass spectrometer operated in both positive and negative ion modes. Raw data were processed using MSDIAL software (version 4.70). For metabolite identification, accurate mass, retention time, and MS/MS spectra were matched against the mzCloud, MassBank, and METLIN databases; according to the Metabolomics Standards Initiative (MSI), the annotations were mainly assigned as Level 2 (putatively annotated compounds) [46,47].

For statistical analysis, a total of 889 features were initially detected. Features with more than 20% missing values were removed, and the remaining missing values were imputed by half of the minimum positive value for each sample. Data were normalized by the internal standard and subsequently subjected to unit variance scaling (UV) preprocessing. PCA and Orthogonal Partial Least Squares Discriminant Analysis (OPLS-DA) were performed. For univariate statistical analysis, *p*-values were calculated using Student’s *t*-test. Differential metabolites were identified based on VIP ≥ 1, |log_2_(Fold Change)| ≥ 1, and FDR < 0.05 (calculated using the Benjamini–Hochberg method). KEGG pathway enrichment analysis was conducted using Fisher’s Exact Test, with significance set at FDR < 0.05 [48,49].

### 4.5. Targeted Hormonal Metabolomic Analysis

Targeted hormone metabolomics technology was employed to quantitatively analyze endogenous auxins, gibberellins, and abscisic acid related metabolites in peel tissues. For each sample, 50 mg of powdered material was extracted using 350 µL of a methanol:water:formic acid (15:4:1, *v*/*v*/*v*) solution containing internal standards. Following vortexing, ultrasonication, and centrifugation, the supernatant was concentrated and prepared for both non-derivatization and BPTAB/TEA derivatization. LC-MS analysis was performed using an LC-30A UHPLC system coupled with a Sciex 6500+ triple quadrupole mass spectrometer in multiple reaction monitoring (MRM) mode. A Waters ACQUITY Premier HSS T3 column was utilized with a mobile phase of 0.04% formic acid in water and acetonitrile, at a flow rate of 0.3 mL/min and a column temperature of 40 °C. The quantification of metabolites was conducted using MultiQuant software (version 3.0.3; SCIEX, Framingham, MA, USA) with internal standard calibration, achieving a coefficient of determination (R^2^) exceeding 0.99 for all standard curves. Differential hormone metabolites were identified based on |log_2_(Fold Change)| ≥ 1 and *p*-value < 0.05. K-means clustering and radar charts were employed for visual representation of accumulation patterns [50,51,52].

### 4.6. Multi-Omics Integration Analysis

To investigate the regulatory relationships between transcriptomic and metabolomic changes, a multi-omics integrative analysis was conducted. To ensure statistical validity given the limited biological replicates (*n* = 3) and the high dimensionality of the data, a strict variable filtering and dimensionality reduction strategy was applied before entering the CCA. Specifically, we first extracted DEGs and DEMs that were significantly enriched in the core KEGG pathway (Plant hormone signal transduction) with FDR < 0.05. After filtering, 368 representative DEGs and 3 DEMs were retained for subsequent analysis. To further reduce dimensionality and avoid overfitting, PCA was performed on the filtered gene and metabolite matrices to extract the core components (PC1 and PC2). CCA was then employed to evaluate the synergistic variations between these filtered DEGs and DEMs. For transcriptomics and targeted hormone metabolomics, the Pearson correlation coefficient was calculated to evaluate the relationship between gene expression levels and the abundance of targeted hormone metabolites. To reduce instability caused by small sample sizes, we focused only on the hormone-related genes and metabolites (from the filtered subset mentioned above), and only pairs with a strong correlation (|r| > 0.8) and a significant *p*-value (*p* < 0.05) were retained. A nine-quadrant correlation plot was generated to visualize the distribution of gene–metabolite pairs based on the strength of their associations. Principal component loadings analysis was performed to identify the dominant variables driving the separation of components in the combined transcriptomic and targeted hormone dataset. The correlation network was constructed and visualized using Cytoscape software (v3.9.1) and the R package “igraph”, where nodes represent genes or metabolites, and edges reflect significant Pearson correlations (|r| > 0.8, *p* < 0.05) [53,54].

### 4.7. Real-Time Quantitative PCR (RT-qPCR)

To validate the transcriptome data, real-time quantitative PCR was performed. Total RNA was extracted from the pericarp of wild-type and mutant *K46* siliques at 20 and 30 DAF using a plant RNA extraction kit, with three independent biological replicates per sample (each biological replicate was pooled from three individual plants). One to two micrograms (1–2 µg) of total RNA were utilized to synthesize the first strand of complementary DNA (cDNA) according to the instructions provided by the RevertAid RT Kit (Thermo Scientific, Waltham, MA, USA). Gene-specific primers were designed using Primer Premier 5.0 software, with the sequences provided in Supplementary Table S5. The amplification efficiency of each primer pair was tested using standard curves and confirmed to be within 90–110%. qRT-PCR was conducted using SYBR Green Premix Ex Taq (Takara, Japan) on the Quant Studio™7 Flex Real-Time PCR System (Applied Biosystems, Carlsbad, CA, USA) [55]. Each reaction mixture (10 µL) contained 5 µL of SYBR Green mix, 0.2 µM of each primer, and 2 µL of diluted cDNA. The amplification program was as follows: pre-denaturation at 95 °C for 3 min, denaturation at 95 °C for 15 s, and annealing at 60 °C for 30 s, for a total of 40 cycles. After each run, a melt curve analysis was conducted to confirm the specificity of the amplification products. The *B. napus ACTIN7* gene (*BnaA03g55890D*) served as a reference control [56]. To validate its stability across genotypes and developmental stages, raw CT values for *ACTIN7* were analyzed using the BestKeeper method, and the results are provided in Table S7. For statistical analysis, the average CT value of three technical replicates for each biological replicate was used. The ΔCt values were calculated and subjected to unpaired Student’s t-tests. The relative expression levels of the genes were calculated using the 2^−ΔΔCT^ method. All data are presented as mean ± SD, and statistical significance was set at *p* < 0.05 [57].

## 5. Conclusions

This study presents the *BnMYB46* knockout mutant *K46*, created using CRISPR/Cas9 technology, and systematically reveals the critical mechanisms by which this gene regulates the normal development of siliques through a combination of cytological microscopy observations, transcriptomics, non-targeted metabolomics, and targeted hormone metabolomics analyses. Our research primarily concludes the following key points: The loss of *BnMYB46* leads to deep folding of the epidermal cells in the silique and severe inward compression and collapse of the underlying parenchyma cells, resulting in compromised cell wall structural integrity and ultimately manifesting as a severe short silique phenotype. Multi-omics combined analysis confirms that during the crucial developmental stage of silique elongation (20 DAF), an imbalance in the homeostasis of early plant hormone signaling—particularly auxin and gibberellin—serves as a pivotal upstream event triggering the aforementioned cellular defects. This hormonal disruption collaboratively contributes to the loss of cellular support and structural support capacity in *K46* by disturbing downstream cell wall synthesis and starch/sucrose metabolic pathways.

## Data Availability

The raw reads obtained from the sequencing are stored in the China National Center for Bioinformation database, under accession number CRA048068 that are publicly accessible at https://ngdc.cncb.ac.cn/ (accessed on 10 August 2026).

## Supplementary Materials

The following supporting information can be downloaded at: https://www.mdpi.com/article/10.3390/ijms27188349/s1.
