# Supplementary material for: Transcriptomic and Metabolomic Investigation of Cell Wall Remodeling and Hormonal Signaling Dysfunction in Short Silique Mutant Rapeseed
Source: Int J Mol Sci. 2026 Sep 19;27(18):8349. doi: 10.3390/ijms27188349 (PMC13607505; doi:10.3390/ijms27188349)
Supplement: Supplementary file 1 [file ijms-27-08349-s001.zip › Supplement pictures.pdf]

Supporting Information

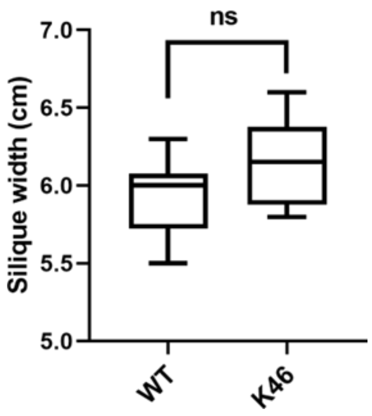

Figure S1. Comparison of silique width between WT and *K46*. Data are presented as mean ± SD (n = 5 biological replicates, with 10 siliques measured per replicate). Statistical significance was determined using an unpaired Student's *t*-test (ns, not significant; P > 0.05).

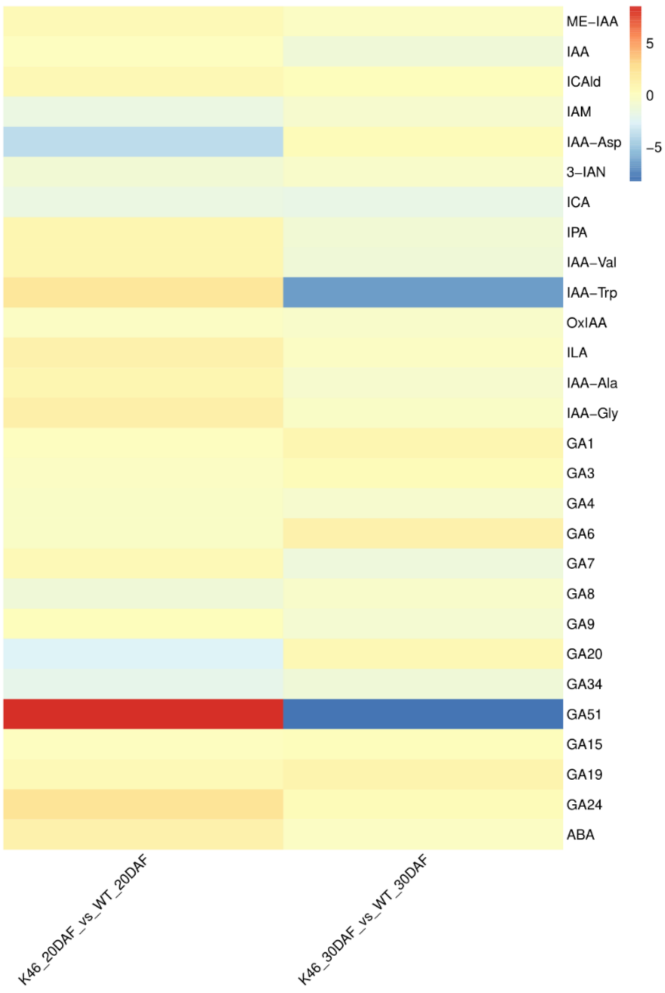

Figure S2. Heatmap showing the accumulation changes (log_2FC) of selected IAA, GA, and

ABA related DEMs in *K46*_20DAF_vs_WT_20DAF and *K46*_30DAF_vs_WT_30DAF comparison groups.

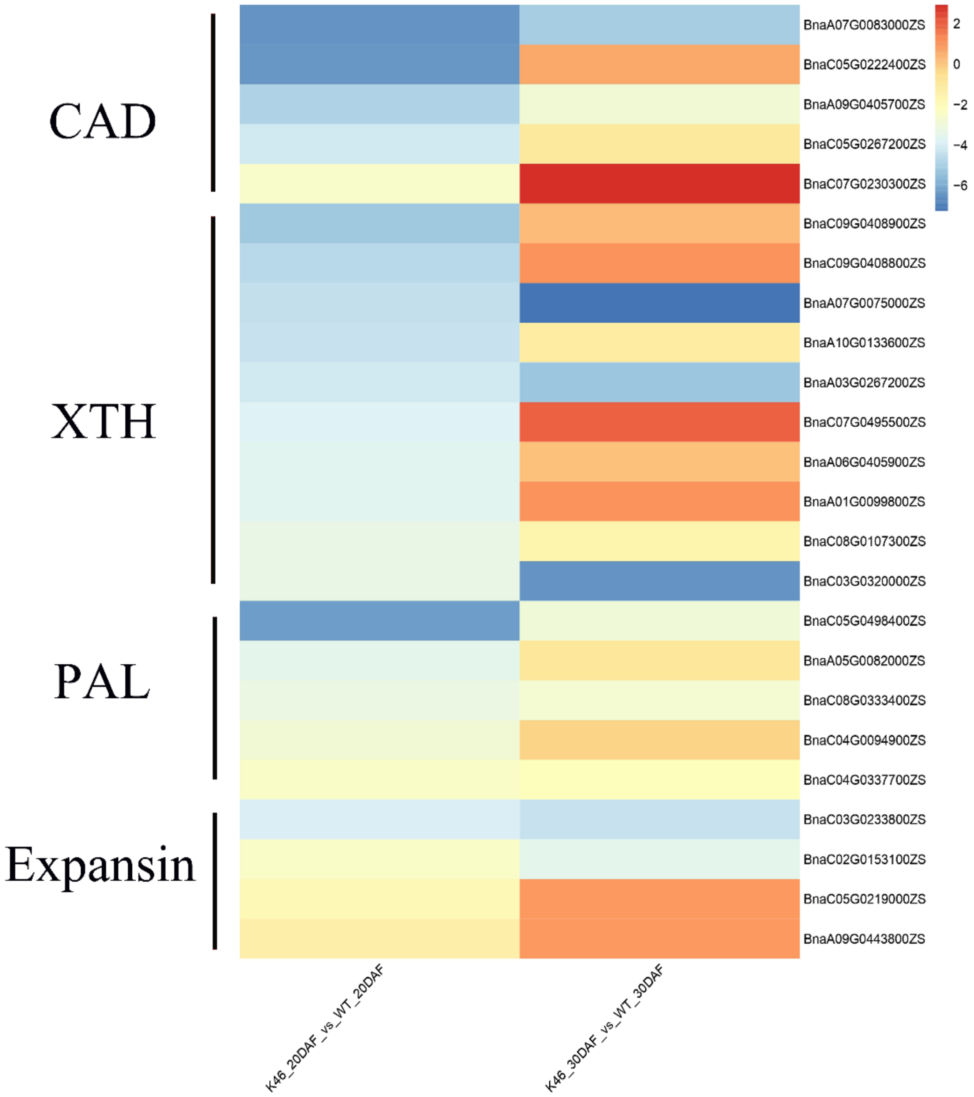

Figure S3. Expression patterns of cell wall-related DEGs in *K46* compared to WT. The heatmap shows the expression changes (log_2FC) of genes involved in cellulose biosynthesis, hemicellulose metabolism (XTH), lignin pathways (CAD, PAL), cell-wall loosening (Expansin), and cell-wall modification at 20 and 30 DAF. Red and blue colors indicate up- and down-regulation, respectively. Asterisks indicate significant differential expression (FDR < 0.05).
